# Zirconium-Porphyrin PCN-222: pH-responsive Controlled Anticancer Drug Oridonin

**DOI:** 10.1155/2018/3249023

**Published:** 2018-12-04

**Authors:** Xin Leng, Hongliang Huang, Wenping Wang, Na Sai, Longtai You, Xingbin Yin, Jian Ni

**Affiliations:** ^1^School of Chinese Materia Medica, Beijing University of Chinese Medicine, Beijing 102488, China; ^2^Beijing Research Institute of Chinese Medicine, Beijing University of Chinese Medicine, Beijing 100029, China; ^3^State Key Laboratory of Separation Membranes and Membrane Processes, Tianjin Polytechnic University, China; ^4^National Center for International Joint Research on Membrane Science and Technology, Tianjin Polytechnic University, China; ^5^School of Pharmacy, Inner Mongolia Medical University, Hohhot 010110, China

## Abstract

Drug delivery carriers with a high drug loading capacity and biocompatibility, especially for controlled drug release, are urgently needed due to the side effects and frequent dose in the traditional therapeutic method. Guided by nanomaterials, we have successfully synthesized zirconium-based metal−organic frameworks, Zr-TCPP (TCPP: tetrakis (4-carboxyphenyl) porphyrin), namely, PCN-222, which is synthesized by solvothermal method. And it has been designed as a drug delivery system (DDS) with a high drug loading of 38.77 wt%. In our work, PCN-222 has achieved pH-sensitive drug release and showed comprehensive SEM, TEM, PXRD, DSC, FTIR, and N_2_ adsorption-desorption. The low cytotoxicity and good biocompatibility of PCN-222 were certificated by the in vitro results from an MTT assay, DAPI staining, and Annexin V/PI double-staining even cultivated L02 cells and HepG2 cells for 48h. Furthermore, Oridonin, a commonly used cancer chemotherapy drug, is adsorbed into PCN-222 via the solvent diffusion technique. Based on an analysis of the Oridonin release profile, results suggest that it can last for more than 7 days in vitro. And cumulative release rate of Ori at the 7 d was about 86.29% and 63.23% in PBS (pH 5.5 and pH 7.2, respectively) at 37°C. HepG2 cells were chosen to research the cytotoxicity of PCN-222@Ori and free Oridonin. The results demonstrated that the PCN-222@Ori nanocarrier shows higher cytotoxicity in HepG2 cells compared to Oridonin.

## 1. Introduction

In the past two decades, microporous metal−organic frameworks (MOFs), combined by different metal ions or clusters and organic ligands [1] giving rise to a crystalline structure, were widely used in gas adsorption and separation [2], supercapacitor electrode [3, 4], catalysis [5], waste-water purification [6], fluorescence detection [7], magnetism [8], etc. Recently, MOFs have an exhibited great potential in the biomedical domain especially as drug delivery carriers to control delivery of target drugs due to its several promising capabilities [9]. MOFs have an irresistible advantage as favorite drug carrier, such as extra-high porosity and surface areas [10], tunable and tailorable particle size [11], designable host-guest interaction [12], multiple topologies [13], modifiable chemical surface [14], and being stable enough under biological conditions [15, 16]. Based on the above research, a variety of novel drug delivery systems (DDS) can be designed, such as high loading capacity of different guest molecules [17], tumor targeted treatment [18], drugs release in response to external stimuli such as pH, temperature, light irradiation, and redox reagents [19–22]. Therefore, MOFs can improve drugs solubility [23], change the pharmacokinetics of drugs [24, 25], prolong circulation time [26], significantly enhance the antitumor effect [27], minimize the dosage, and reduce toxic side effects [28].

**Figure 1:**
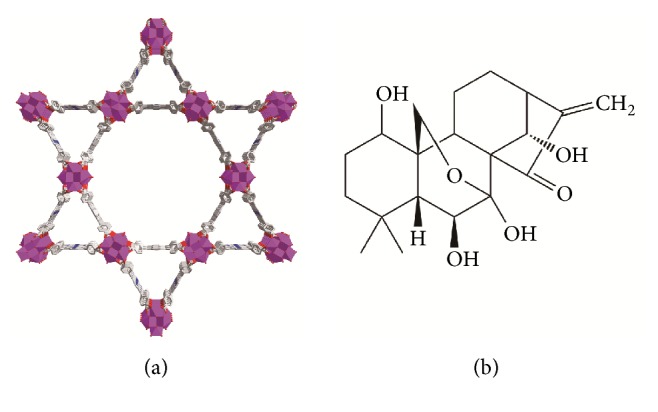
Structural diagram. (a) schematic illustration for the construction of PCN-222 and (b) the chemical structure of Oridonin.

The Zr_6_ cluster is thought to be a perfect building block to synthesize the mixed-linker MOFs, because of their intrinsic open frameworks, superior stability, and adjustable connectivity [29]. Therefore, Zr-MOFs, as a class of new porous skeleton material, have gained wide applications due to their ultrahigh surface areas and being more stable in the aqueous phase than general Fe/Zn/Cu/Cd-based MOFs [30]. Porous coordination network (PCN-222) is a kind of MOF formed by zirconium chloride octahydrate and meso-tetra (4-carboxyphenyl) porphyrin; nanoporous structure is shown in Figure 1(a). The original design target of preparing PCN-222 (Zr_6_(*μ*-OH)_8_(OH)_8_(CO_2_)_8_) is to be as biomimetic catalysts [31], and then it is applied to the label-free detection of a phosphoprotein (*α*-casein) [32]. In biomedical applications, Zr-MOFs can form Zr−O−P bonding while maintaining its integrity frameworks; therefor, Zr-MOFs have high affinity with organophosphorus species [33]. Although several biocompatible porous MOFs were explored as drug delivery, Zr_6_ and porphyrin-based MOFs remain to be reported.

Oridonin (Ori) is a well-known ent-kaurene tetracyclic diterpenoid compound (Figure 1(b)) isolated from the Chinese medicinal herb Rabdosia rubescens [34] with various pharmacological actions. Ori has raised wide attention, in recent years, because of its conspicuous antitumor activity, such as gastric cancer [35], multiple myeloma [36], triple negative breast cancer [37, 38], hepatocellular carcinoma [39], breast cancer [38], prostate cancer [40], oral cancer [41], and osteosarcoma [42]. Pharmacological study shows that the chief anticarcinomatous mechanism of Ori lies in the following: (1) significant antimutagenic effect, (2) inhibiting sodium pump activity, (3) antiangiogenic activity, (4) inducing cell apoptosis [34, 43, 44], etc. However, the Ori with poor solubility, instability in chemical property, short biological half-life, and active group (*α*-methylene-cyclopentanone) was easily deactivated, which severely prevents its clinical applications [45]. Therefore, it was necessary to prepare a kind of sustained-release preparations to prolong the half-lifetime, increase the bioavailability, and reduce the side effect of Ori. But so far, research on Ori delivery material is rare. Studies on sustained-release or controlled-release preparations of Ori were restricted to the most familiar carriers, such as galactosemia chitosan [46], HP-*β*-cyclodextrin [47], poly(lactic-co-glycolic) acid [48], and graphene oxide [49]. Although drug stability and efficacy improved by the above pharmaceutical methods, as we know, drug loading capability of organic carrier is disappointed and inorganic carrier is difficult to be degraded and removed from body [50]. One of the important research directions is to develop or synthesize a suitable drug carrier of Ori.

Based on the above thought, we have successfully synthesized a biocompatible Zr-based nanoscale MOFs (PCN-222) with unique advantage, high load dose, nontoxicity, biocompatibility, pH-sensitive release, etc. We aim to prepare a PCN-222@Ori sustained-release and controlled-release drug delivery system by using PCN-222 load Ori. To the best of our knowledge, PCN-222 is the first MOF that carries Oridonin.

## 2. Experimental Section

### 2.1. Synthesis and Characterization

The synthesis of PCN-222 was realized by dissolving meso-tetra (4-carboxyphenyl) porphyrin (TCPP, 0.4g) and zirconium chloride octahydrate (ZrOCl_2_·8H_2_O, 2.0g) in 500 mL of dry N, N-dimethylformamide (DMF), and 300 mL of formic acid. The mixture was placed in a round bottom flask equipped with a condenser and was kept stirring and heated for three days at 408 K under air. The mixture was returned to room temperature. Dark red solid was recovered by filtration. In order to remove unreacted starting ligands, inorganic species, as-synthesized PCN-222 (~100mg) samples were immersed into 100mL DMF with 3mL of 4M HCl at 120°C for 12 h. After cooling to room temperature, the supernatant was carefully decanted and washed with DMF and acetone for three times. Fresh acetone was subsequently added, and the sample had a right to stay for 8h to exchange and remove the nonvolatile solvates (DMF) and this process was repeated six times. After removal of acetone by decanting, the sample was activated by drying under vacuum for 6h.

The synthesized PCN-222 was characterized by scanning electron microscopy (the samples that need checking were fixed on the aluminum sample column with carbon conductive tape and observed after the gold sputtering treatment 2.5min, SEM, Hitachi S-4800, Japan), transmission electron microscopy (the samples were mixed by anhydrous alcohol, a drop of the prepared solution was transferred to the carbon film coated grids for overnight drying, TEM, JEM-1230, Japan), N_2_ adsorption-desorption at 77K (Micromeritics ASAP 2020, USA), powder X-ray diffraction (Cu-K*α*, *λ* =1.541nm, 40Kv/40mA, XRD, Rigaku Ultima IV, Japan), and enzyme-sign instrument of multiple function (the PCN-222 was mixed by high purity water, transferred into 96-well plate (plate, clear bottom black with lid), and its fluorescence property was determined, Molecular Devices SpectraMax i3X, USA).

### 2.2. Cell Culture, Cytotoxicity Assay, Imaging, and Annexin V/PI Double-Staining Assay

Human hepatoma cells (HepG2, Jenniobio Biotechnology, China) and human normal hepatocyte cells (L02, Jenniobio Biotechnology, China) were incubated in a humidified incubator (37°C, 5 % CO_2_) for two or three days employing DMEM (Coning, USA) with 10 % fetal bovine serum (FBS, China) and 1 % penicillin-streptomycin solution (USA). The cytotoxicity of PCN-222 at various concentrations (0, 10, 20, 40, 80, and 160 *μ*g/mL) was evaluated through standard MTT assays used L02 cells. And the anticancer activity of free Ori and PCN-222@Ori was assessed by HepG2 cells. After that, the MTT solution with a concentration of 5 mg/mL was added to each well and incubated for another 4 h. Culture supernatant was removed and dimethyl sulfoxide (DMSO, 150 mL) was added to each well, and then the plate was shaken for 10 min. The absorbance of each well was read at *λ*= 570nm in a microplate reader (Thermo, Multiskan GO, USA). All tests were repeated for three times and the data were averaged, having the exact same protocol to evaluate the antitumor activity of the Ori (0, 10, 15, 20 *μ*g/mL) and PCN-222@Ori. In order to further evaluate the biocompatibility of PCN-222, the leakage rate of lactate dehydrogenase (LDH) was assayed for the evaluation of the cell membrane intact. After the L02 cells were treated with PCN-222 (0, 10, 20, 40, 80, and 160 *μ*g/mL) for 48, the supernatant was collected, and LDH activity was determined with a commercial kit (Beyotime Biotechnology, China) in accordance with the manufacturer's instructions. The experiments were performed in triplicate. Besides, L02 cells were seeded with PCN-222 in a 6-well plate and incubated for 48 h. For each well, culture supernatant was removed and fixed by using 4% paraformaldehyde (500 mL) for 10 min at room temperature. Apart from that, the fixed cells were washed two times by using 0.5 mL PBS and stained with a DAPI (Beyotime Biotechnology, China) solution for 5 min to visualize nuclear DNA. Thereafter, the cells were washed three times with PBS (1.0 mL) and nuclear changes were analyzed using confocal laser scanning microscopes (Olympus, IX73, Japan). What is more, an Annexin V-FITC detection kit (Beyotime Biotechnology, China) was used to exam the cell apoptosis [51, 52] by flow cytometry (BD FACSCanto II, USA).

### 2.3. Preparation and Characterization of PCN-222@Ori

The PCN-222@Ori pH-sensitive nanoparticles were prepared by the solvent diffusion technique (QESD). Ori (400.48 mg, Nantong Feiyu Biological Technology Co., Ltd., China) was dissolved in methanol and made into 8.0 mg/mL standard solutions. The dried PCN-222 (3 mg) with a different amount of the standard solution in Xilin bottles was subject to magnetic stirring at room temperature. The orthogonal design L_9_ (3^3^) was implemented to ensure the optimum process. And we studied how the ratio of drug to PCN-222 (A), mixing time (B), and the amount of solvent (C) influenced the process of loading. SEM, TEM, XRD, Fourier transform infrared spectroscopy (studied by the KBr method, FTIR, Thermo Fisher Nicolet6700, USA), and differential scanning calorimetry (the samples were placed on a flat-bottomed aluminum plate and tested at 40-300°C, heating rate of 10°C/min, and nitrogen flow rate of 50 mL/min, DSC, Mettler-Toledo Stare, Switzerland) were used to describe its characteristics.

### 2.4. Measurement of the Drug Loading Capacity

PCN-222@Ori was precipitated through methanol cleaning twice and centrifuging (9500 rpm, 15 min) after 2d, 3d, or 4d. The supernatant of methanol was collected and its concentration of Ori was determined by HPLC (Methanol: Water = 55: 45, *λ* = 295 nm) via an Agilent Zorbax SB-C_18_ system. And the formulae of PCN-222 loading capacity (LC) is as follows: LC%=(M_0_-M_1_)/M×100% (M_0_ and M_1_ are the initial amount and the final amount of Ori (mg) in the system, respectively. M denotes the amount of PCN-222@Ori (mg)).What is more, to get the exact amount of Ori in PCN-222@Ori, the ^1^H-NMR spectra (Bruker 800MHz AVANCE III HD with a cryoprobe) of alkaline-digested PCN-222@Ori are analyzed. The relevant signals of TPCC ligands are then integrated against those of Ori, resulting in peak radios of about 1:0.075, implying the mole ratios of TCPP/Ori is about 1:0.6.

### 2.5. In Vitro Ori Release Profile

For the determination of the impregnated drug molecule release from the PCN-222@Ori, an in vitro cumulative Ori release study was performed in various pH environments. A semipermeable dialysis bag diffusion technique (dialysis bag, MW3400, MD34 mm, USA) was implemented to evaluate the cumulative drug release. Firstly, approximately 10mg of PCN-222@Ori was dispersed at pH7.2 (100mL) followed by incubation at 37°C, and then exactly the same experimental procedure was executed at pH5.5 (100mL). At the given time, 3 mL of the digestion liquor was collected, and the equivalent volume of fresh PBS was added. And the digestion liquor was filtered through a 0.45*μ*m polytetrafluoroethylene membrane filters for Ori analysis. The cumulative percentage of Ori release was determined by using HPLC via an Agilent Zorbax SB-C18 system in comparison with a standard curve of free Ori (Y=26292X– 8127, r^2^ = 0.999, from 0.06*μ*g/mL to 84.0 *μ*g/mL) at regular predetermined time intervals. The release mechanism was analyzed by zero-order, first-order, Higuchi, Weibull, and Ritger-Peppas equations.

## 3. Results and Discussion

### 3.1. PCN-222 Nanocomposites Preparation and Characterization

**Figure 2:**
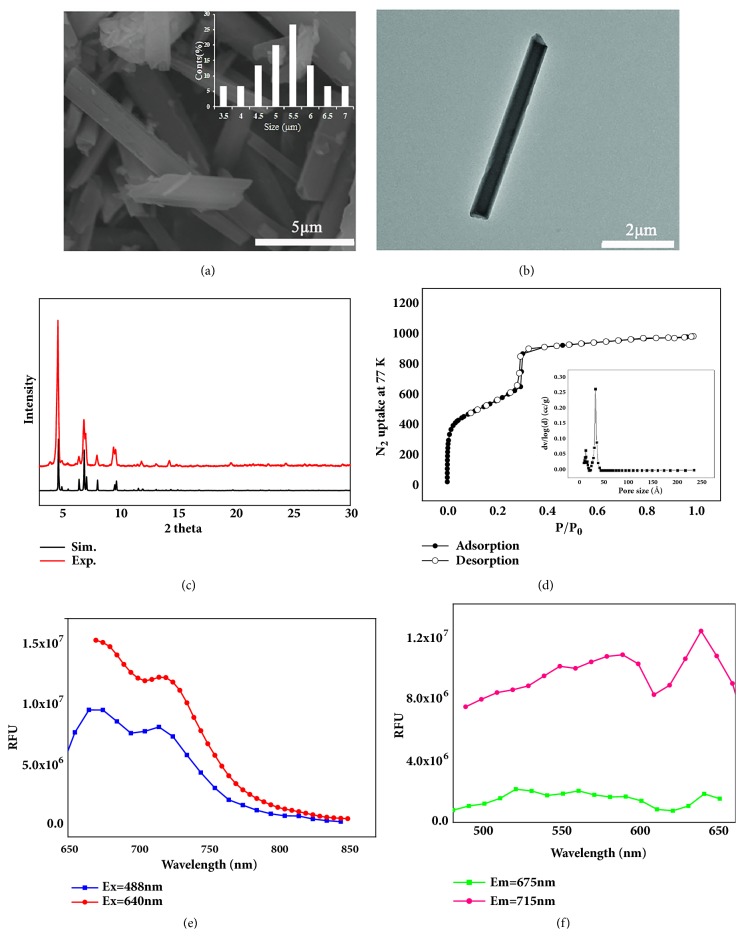
Surface topography and structural analysis of PCN-222: (a) SEM, (b) TEM, (c) PXRD spectra, (d) the N_2_ adsorption-desorption isotherms at 77 K for PCN-222, (e) emission wavelength spectrum, and (f) excitation wavelength spectrum of PCN-222 (dispersion in high purity water).

As large pore kind of Zr-MOF, PCN-222 can be synthesized in aqueous phase under mild conditions and is a promising candidate for encapsulating anticancer drugs. Dark violet rod-like crystals of PCN-222 were obtained via solvothermal reactions. SEM (Figure 2(a)) and TEM (Figure 2(b)) images indicated that PCN-222 had a diameter of approximately 5.27*μ*m, and these NPs show a perfect drug loading capacity [53]. The XRD was performed to analyze the powder purity of PCN-222 at room temperature, and the main crystalline peaks are obvious at 5.24°, 7.24°, and 9.80°, which well agree with the simulated peak pattern (Figure 2(c)). The porous structure of PCN-222 was explored by N_2_ adsorption-desorption isothermals at 77 K. The typical type IV isotherm of PCN-222 exhibits a steep increase at the points of P/P_0_=0.05 and 0.3, respectively, suggesting that both micropore and mesopore existed in the MOF (Figure 2(d)). The BET specific surface area of PCN-222 was calculated to be 2476 m^2^/g and the pore volume was 1.53 cm^3^/g. The pore size distributions based on DTF method show that the pore size is 1.2 nm and 3.2 nm, respectively, similar to that reported in the previous literature [54]. The large specific surface area and pore volume of the sample provide the possibility for high loading of the drug. And the fluorescence of PCN-222 was determined at maximum excitation/emission wavelengths as well as 675/640nm (Figures 2(e) and 2(f)) indicating that the fluorescence of PCN-222 can avoid biological autofluorescence and has good tissue penetrability [55].

### 3.2. Evaluation of Nanosafety of PCN-222 Designed for Drug Delivery

**Figure 3:**
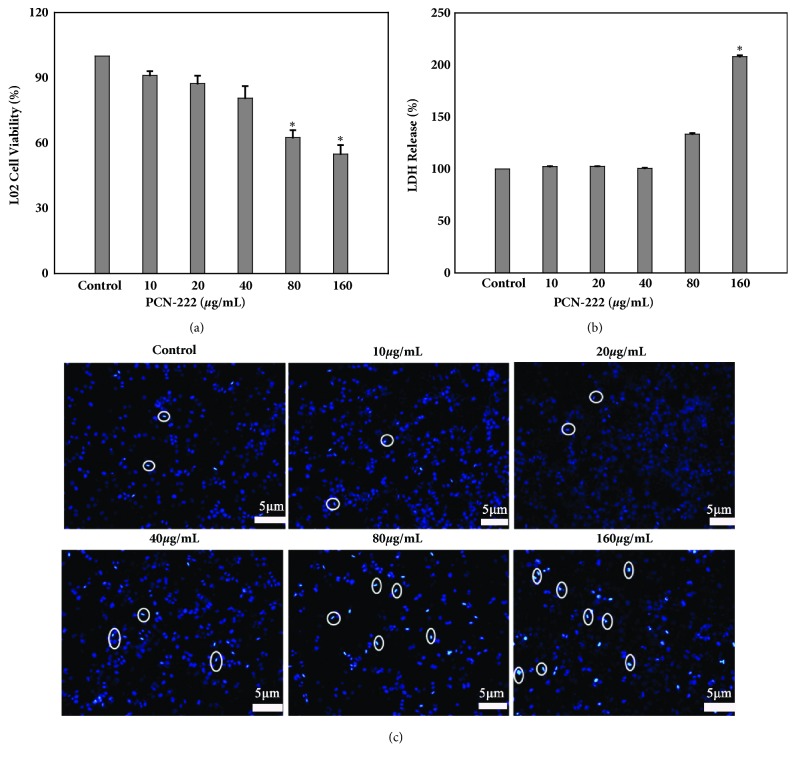
Effects of PCN-222 on cell viability and morphology. (a) MTT assay data were presented as mean ± SD of viability % of three independent experiments. (b) LDH assay was used to assess cell membrane damage and results were presented as mean ± SD of three independent experiments. (*∗*p< 0.05 versus control). (c) L02 cells nuclear morphology was evaluated using DAPI staining (the circle markers represent the apoptotic cells).

**Figure 4:**
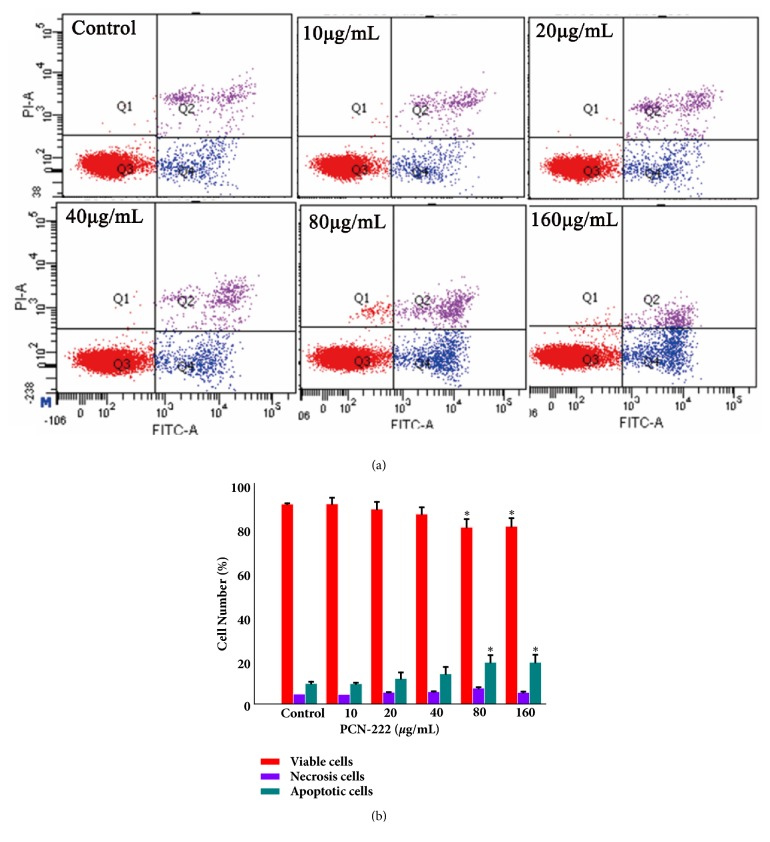
Effects of PCN-222 on apoptosis in L02 cells. (a) Flow cytometry detection of apoptosis with FITC-Annexin V/PI double staining. (b) The percentages of viable, early apoptosis, and necrosis cells of L02 cells after incubation with different concentrations of PCN-222 for 48 h. The data are expressed as means ± SD from three independent experiments (*∗* p < 0.05 versus control).

For the delivery of nanomaterials with large specific surface area, it easy to enter and deposit into the liver cells. The cytotoxicity of the PCN-222 in L02 cells was determined by the MTT assay. Cells were treated with different concentrations (0–160 *μ*g/mL) for 48h, and the result (Figure 3(a)) showed that there was no significant (P>0.05) effect on L02 cell proliferation under 40 *μ*g/mL when compared with vehicle controls indicating that the PCN-222 is less toxic toward L02 cells. Leakage of LDH is a sign of cell membrane damage. The results showed that leakage of LDH occurring in L02 cells have few effects after treatment with PCN-222 under concentration of 80 *μ*g/mL (Figure 3(b)). DAPI staining (Figure 3(c)) observed that PCN-222 treated cells have suitable morphology with an intact nucleus and only at extremely high concentration may induce condensation of chromatin and nuclear fragmentation. In addition, following treatment with PCN-222 for 48 h, the percentage of viable cells had insignificantly changed below 40 *μ*g/mL. Furthermore, the percentage of early apoptosis cells increased only from 4.80 % ± 0.56 to 13.63 % ± 2.16, and necrotic and late apoptotic cells almost do not increase as PCN-222 is increased (Figures 4(a) and 4(b)). In short, PCN-222 might cause the cell apoptosis, damage cell membrane, and split nuclear under ultimate high concentration, but it has good biosafety and cell biocompatibility under 40 *μ*g/mL. Those studies clearly indicate that PCN-222 are nontoxic toward the cell under 40 *μ*g/mL.

### 3.3. Optimal Loading Process and Evaluation of PCN-222@Ori

**Figure 5:**
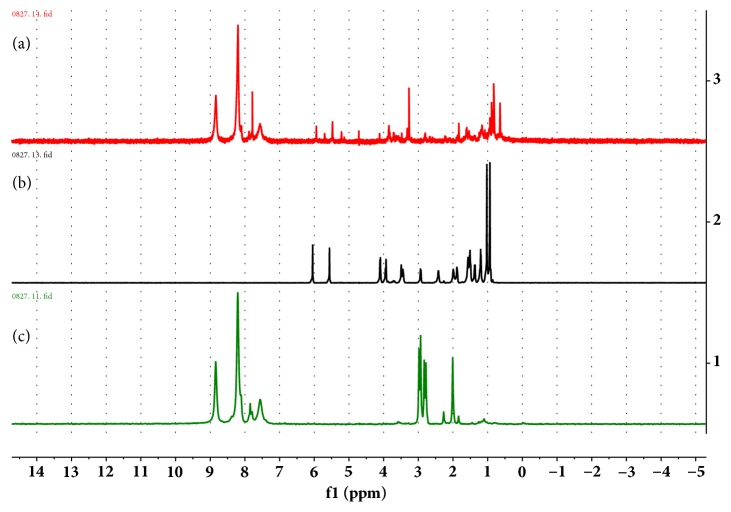
^1^H-NMR spectra of (a) alkaline-digested PCN-222@Ori, (b) alkaline-digested Ori, and (c) PCN-222 in KOH/D_2_O solution.

**Figure 6:**
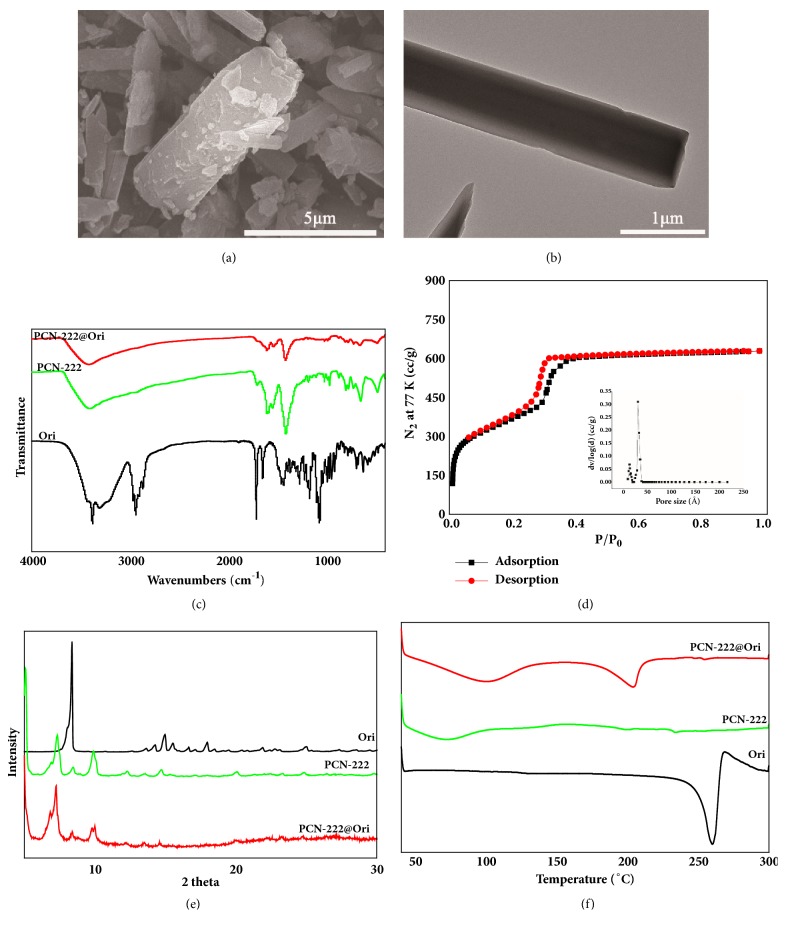
Surface topography and structural analysis of PCN-222@Ori: (a) SEM, (b) TEM, (c) FTIR, (d) the N2 adsorption-desorption isotherms at 77 K, (e) XPRD, and (f) DSC.

By using the L_9_(3^4^) orthogonal table, three factors such as rate of Ori to PCN-222 (A), mixing time (B), and the amount of solvent (C) were selected to be optimized. The drug loading rate reached up to 38.77 wt%, under optimized conditions: PCN-222: Ori: Methanol (1: 3: 1), with magnetic stirring for 4 days. To further confirm the existence of the drug Ori in PCN-222@Ori, the ^1^H-NMR spectra of alkaline-digested PCN-222@Ori were analyzed in KOH/D_2_O solution, as shown in Figure 5(a). To identify the peak of the TCPP ligand and Ori molecule solution, the ^1^H-NMR spectra of the alkaline-digested PCN-222 and Ori in KOH/D_2_O solution were also provided in Figures 5(b) and 5(c). Obviously, the peak of Ori can be observed in alkaline-digested PCN-222@Ori solution. During drug loading, PCN-222 can preserve the original structure while Ori entered the void structure, shown in SEM (Figure 6(a)) and TEM (Figure 6(b)) images. The spectra of PCN-222@Ori were consistent with the spectra of PCN-222, shown in Figure 6(c). The intensity of the absorption peak and the small changes in the position indicate that there is some interaction between PCN-222 and Ori. To gauge the porosity of the PCN-222 following Ori installation, N2 adsorption-desorption experiments were conducted at 77 K; the obtained isotherms of PCN-222@Ori are shown in Figure 6(d). The BET specific surface area of PCN-222@Ori was calculated to be 1258 m^2^/g and the pore volume was 0.97 cm^3^/g. Both sets of isotherms indicate decrease of porosity following Ori introduction, and the pore size is 1.2 nm and 3.2 nm, respectively, by the pore size distribution plots. It is noteworthy that the BET specific surface area and the pore volume of PCN-222 are reduced, so the drug is loaded inside the MOF pore rather than sitting on the surface of the MOFs. From the result of X-ray diffraction pattern (Figure 6(e)) and DSC spectra (Figure 6(f)), the absorption peak of PCN-222@Ori completely disappeared after taking the drug, indicating that the Ori was distributed in PCN-222 with amorphous state.

### 3.4. pH-Responsive Release Profile

**Figure 7:**
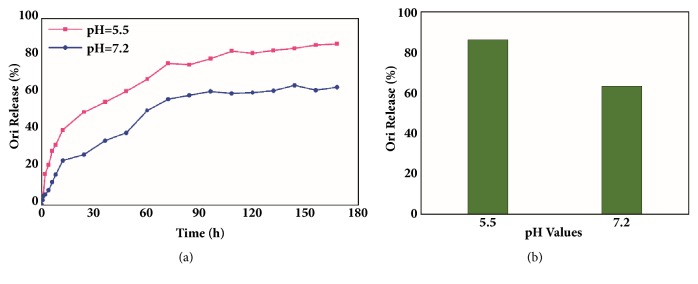
In vitro Ori release profiles of the PCN-222@Ori at different pH values. (a) The release curve and (b) the total amount of drug release.

The pH value in tumor and inflammatory tissues tends to be more acidic (pH 6.0–7.0) than that in blood and healthy tissue (pH 7.4), just as was said by Valeria De Matteis [56]: this important factor may be used to design pH-responsive nanosystem targeted at tumor therapy. Thus, we can design nanocarriers that are sensitive to pH signals to trigger selective drug release in cancer cells. In this study, the Ori release profiles of PCN-222@Ori were explored at two different pH values (pH 7.2 and pH 5.5). And PCN-222@Ori at acidic condition (pH 5.5, 86.29%) showed a significantly higher drug release rate than those at the neutral condition (pH 7.2, 63.30%) in 168 h, as is shown in Figure 7. The releasing characteristic parameter was explored by different mathematical model to explain the mechanism of drugs release, and the fitting results are shown in Table 1. The result of the study on the mechanism of drug release showed that in vitro drug release was fitted to Weibull and mechanism of release was diffusion. The fitted equations at pH 5.5 and pH 7.2 are lnln(1/(1-R_t_)) = 0.6384lnt – 2.4694 (R^2^=0.977) and lnln(1/(1-R_t_)) = 0.653lnt-3.169 (R^2^=0.987), respectively.

### 3.5. Cytotoxic Effect of PCN-222@Ori on HepG2 Cells

**Figure 8:**
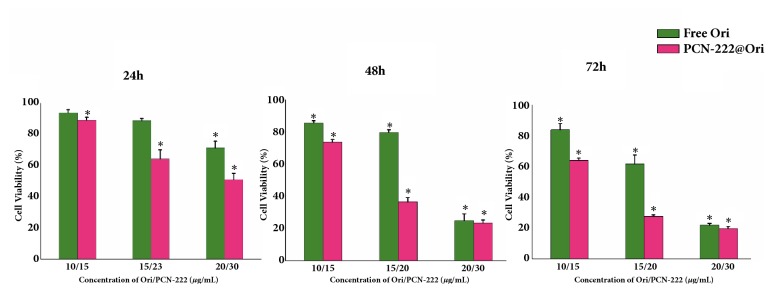
Comparison of the antitumor activity of PCN-222@Ori and free Ori by incubating various concentrations of samples for 24 h, 48 h, and 72 h with HepG2 cells.

Free Ori and PCN-222@Ori were also investigated for the cancer therapy. PCN-222@Ori showed excellent therapeutic efficacy for HepG2 cells as the dosage increased with time prolonged, compared with free Ori. As is shown in Figure 8, after the treatment with free Ori at a concentration of 20 *μ*g/mL for 24 h, 48 h, and 72 h, the cell viability fell from the baseline level to 71%, 25%, and 21%, and after the treatment with PCN-222@Ori, the cell viability decreased to about 51%, 23%, and 19% at the concentration of 20 *μ*g/mL for 24 h, 48 h, and 72 h, respectively. Toxicity of PCN-222@Ori was higher than that of free Ori as well as equal amount of Ori. It is indicated that PCN-222, which we synthesized, and Ori have potential synergistic effect. It is noteworthy that PCN-222 was safe for L02 cells at high anticancer activity. In summary, PCN-222 has been developed as a pH-responsive nanosystem for drug delivery in the cancer therapy.

## 4. Conclusions

The inorganic cluster of PCN-222 (Zr_6_(*μ*-OH)_8_(OH)_8_(CO_2_)_8_) can provide high density of hydroxyl groups, which can bind with Ori molecules via H-bonds interaction, and it owns two types of 1-D channels which provide sufficient space for loading Ori [57]. The isoelectric point pH_pzc_ of PCN-222 is at 7.0-8.0 [58], so the force of H-bonds between PCN-222 and Ori decreases under acidic conditions (Ph 5.5), which is the reason why the release rate of PCN-222 under acidic conditions is faster than that under neutral conditions.

In summary, we have successfully fabricated a PCN-222, which can be used as an ideal drug delivery system. Frameworks with high surface area and suitable pore size are good candidates for Ori loading. What is more, it provides opportunity to reduce the adverse effects of Ori because the system showed a release mechanism. Low cytotoxic activity, efficient drug loading capacity (about 38.77 wt %), and controlled release with a Weibull distribution drug release under different pH values of PCN-222 prove its practical value as a drug carrier. In particular, the pH-responsive release of material confirms the potential applications of PCN-222 as smart drug carriers.

## Figures and Tables

**Table 1:** Fitting curve by different mathematical model under different pH values (Rt: accumulated release rate).

	pH=5.5		pH=7.2	
	Equation	r^2^	Equation	r^2^
zero-order	R_t_ = 0.0047 t + 0.2433	0.811	R_t_ = 0.0039t + 0.1298	0.850
First-order	Ln(1-Rt) = -0.0118 t - 0.2627	0.944	Ln(1-Rt) = - 0.0065t - 0.1379	0.897
Higuchi	R_t_ = 0.0679 t^1/2^ +0.0874	0.952	Rt= 0.055t^1/2^ + 0.0079	0.963
Weibull	lnln[1/(1-R_t_)] = 0.6384lnt - 2.4694	0.977	lnln[1/(1-R_t_)] = 0.653lnt - 3.1688	0.987
Ritger-Peppas	ln (R_t_/R_*∞*_) = 0.4862lnt - 2.4274	0.933	ln (R_t_/R_*∞*_) = 0.5608lnt - 3.1287	0.981

## Data Availability

The original data used to support the findings of this study are available from the corresponding author upon request.
